# Evaluation of biochemical, histopathological, hematological, and genotoxic effects of some indigenous weed plant extracts in albino rats toward a natural and safe alternative to synthetic insecticides

**DOI:** 10.3389/fvets.2026.1694297

**Published:** 2026-02-05

**Authors:** Muhammad Asif Zahoor, Muhammad Kashif Zahoor, Hina Rizvi, Zeeshan Nawaz, Aftab Ahmed, Bushra Munir, Mudassir Hassan, Muhammad Zulhussnain

**Affiliations:** 1 Institute of Microbiology, Government College University, Faisalabad, Pakistan; 2 Department of Zoology, Government College University, Faisalabad, Pakistan; 3 Department of Environmental Sciences and Engineering, Government College University, Faisalabad, Pakistan; 4 Department of Biochemistry, University of Agriculture Faisalabad, Faisalabad, Pakistan; 5 Institute of Chemistry, University of Sargodha, Sargodha, Pakistan; 6 Department of Zoology, Baba Guru Nanak University, Nankana, Pakistan

**Keywords:** *Achyranthes aspera*, blood parameter, *Chenopodium murale*, cypermethrin, enzyme modulation, genotoxicity, histopathology

## Abstract

**Introduction:**

The indiscriminate use of pesticides poses a significant risk to human health and the environment. Plant-based biopesticides offer an alternative to insecticides for integration into insect pest management programs.

**Methods:**

The current study assessed the toxicological effects of leaf extracts from indigenous weed plants, *Chenopodium murale* and *Achyranthes aspera*, in albino rats, *Rattus norvegicus*. The extract-mixed diet was fed in three different doses (100 ppm, 150 ppm, and 250 ppm) for 28 days, while the Cypermethrin insecticide was used as a reference insecticide at the same dose levels. Samples from liver and kidney tissues were collected for histopathological study, and the blood serum was obtained for biochemical assay.

**Results and discussion:**

Histopathological analysis of cypermethrin revealed congestion in the central vein, hemorrhage in hepatic tissues, and necrosis of liver tissues, while kidney tissues showed necrosis of renal tubules, fibrosis, and swelling in Bowman’s capsule. Moreover, hemorrhage was attenuated by degenerated inflammatory cells, edema, and shrinkage, and the rupturing of glomeruli was also observed. Mortality was also recorded at 28th day. In contrast, no physical signs of toxicity and significant alteration in liver and kidney tissues were shown by both plant extracts. Acetylcholinesterase (AChE) and phosphatase enzymes also showed non-significance with plant extracts and significant results with Cypermethrin. Similarly, genotoxicity through the comet assay revealed no changes for either plant. Hematological parameters showed no significant change with plant extracts. Non-significant results revealed that both plant extracts had no difference when compared to the control for all parameters, which indicates that the weed plants are less toxic as compared to Cypermethrin in vertebrates.

**Conclusion:**

These findings suggest that these weed plants have the potential to be used as biopesticides for future integrated pest management (IPM) programs.

## 1 Introduction

Synthetic chemicals are extensively used for quick pest control worldwide. Unlike other conventional control measures, it leads to a rapid eradication of massive insect production (1). However, their excessive use creates a number of environmental concerns, such as biomagnification of pesticides in the food chain and persistence of insecticide resistance in insects. Environmental pollution is considered the most serious issue mankind is currently facing (2, 3). In the larger context, it is a by-product of human activities, i.e., indiscriminate use of pesticides affects living populations either directly or indirectly (4, 5). Being universally and easily available around, pyrethroids are extensively used pesticides with a wide range of applications, i.e., crops, gardens, and homes. Resultantly, there is a considerable risk that these pesticides contaminate the ecosystem. In addition to numerous health concerns, exposure to these pesticides has been linked with a decline in biodiversity, bird species, particularly fish-eating birds, fish, and various other useful insects (2, 3, 6–8). Even very low residual level of synthetic pesticides have been reported to cause disastrous consequences such as brain and nerve damage, liver problems (hepatic fibrosis and jaundice), teratological defects, allergenic sensitization, respiratory diseases (asthma and emphysema), infertility or sterility, blood disorders (blood coagulation defects and anemia) paralysis, impotence, cancer, and various other genetic defects (9–15).

Since excessive use of these synthetic pesticides raises significant environmental and health concerns, this is considered the most pressing issue worldwide. Subsequent exposure to these chemicals is linked to changes in physiological functions, reproductive performance, enzymatic activities, and various other biochemical and hematological parameters. Hence, attention has been focused on the toxicological impact of these insecticides in insects and mammals (13, 16–22).

So far, more than 2,400 plant species have been identified as they have bioactive compounds responsible for their insecticidal properties (19, 23–27). With an increasing trend in the use of plant-based biopesticides, a considerable number of indigenous plants have also been reported for their insecticidal activity (19, 20, 23, 28, 29, 30).

Plant extracts have been demonstrated as alternatives to synthetic pesticides, suggesting that they could be integrated in agriculture sector for insect pest control. Additionally, plant-based bio-pesticides are considered non-toxic to non-target species and, more importantly, they are environmentally safe. We have previously reported that *Achyranthes aspera* and *Chenopodium murale* were effective, particularly against *Drosophila*, house flies, mosquitoes, and stored grain insects, and potentially could be used against a range of insect pests as a controlling agent (19, 20, 23, 28, 30).

Given the efficacy and insecticidal potential of *A. aspera* and *C. murale*, it was aimed to determine whether these plants, when used as biopesticides, are safe for humans and other mammals. Therefore, the albino rat, most commonly used model organism for pharmacology, toxicology, physiology, and pathophysiological research work, was selected in this study to evaluate the toxicological response of *Achyranthes aspera* and *Chenopodium murale*. Cypermethrin is a broad-spectrum synthetic pyrethroid, ranked top across Pakistan and being extensively used against a wide range of insect pests (31). Therefore, this pyrethroid insecticide was selected as the reference treatment during this study.

## 2 Materials and methods

The present study was designed to evaluate the toxic effects of *Achyranthes aspera* and *Chenopodium murale* in albino rats, using pyrethroids Cypermethrin as a reference. The experimental work was performed at the Animal Lab, Department of Pharmacy, and Department of Zoology, Govt. College University Faisalabad.

### 2.1 Collection of plant material

Whole plants of *C. mural* and *A. aspera* were collected from their natural habitat, Faisalabad. The collected plants were cleaned with distilled water, kept under shade at room temperature to dry, and then crushed into small pieces. They were further dried using an oven and converted into fine powder using an electric grinder. Extraction was performed following the methodology described by Sultana et al. (28) with minor modifications. The obtained fine powder (250 g) was dissolved in absolute ethanol (1,000 mL) at a 1:4 ratio (w/v) and was shaken well on a rotary shaker @ 250 rpm for 24 h.

### 2.2 Preparation of crude extracts

The ethanolic plant extracts were filtered using Whatman No. 1 filter paper to obtain a clear extract. Crude extract was obtained from this ethanol filtered extract by concentrating it at 50 °C with the help of a rotary evaporator. The obtained crude extract was stored at 4 °C in a refrigerator until used. The doses of 100, 150, and 250 ppm used in the present study were prepared by diluting the extracts in distilled water (19, 22, 23, 28).

### 2.3 Experimental design

The study was performed for 28 days in 3–4 weeks old albino rats, *Rattus norvegicus* of 100—190 g in weight (13), purchased from the animal house of the University of Agriculture Faisalabad and their rearing was carefully maintained at lab conditions as described by Khalil et al. (21), Khalil et al. (22), and Akhtar et al. (32). Rats were orally administered with different doses (100, 150, and 250 ppm) of plant extracts and cypermethrin (mixed in food), and data were collected at every 7, 14, 21, and 28 days. All the albino rats were weighed before starting the experiment. Cypermethrin and plant extracts were given to albino rats through oral administration with diet on alternate days. The experiment was designed in four main groups, i.e., Control, Cypermethrin, *C. murale*, and *A. aspera*. Three concentrations; 100, 150, 250 ppm were used for three subgroups within each main group of treatment. Each subgroup randomly contained eight (n = 8) albino rats (22, 33). All the experimental procedures adhered to the guidelines of the National Institute of Health (NIH) and OECD standards (TG 407) of 28 days trial study for the care and use of laboratory animals (32, 34). The approval was made prior to the start of work from the Ethical Committee of the Government College University, Faisalabad (416-A, GCUF).

### 2.4 Dissection and samples preservations

The rats were anesthetized and sacrificed at 7, 14, 21, and 28 days. A combination of ketamine (80–100 mg/kg) and xylazine (5–10 mg/kg) intraperitoneally was administered following the protocol of Aguwa et al. (35). Blood samples were collected in well-labeled serum vials containing EDTA solution for biochemical/enzymatic analysis, while the liver and kidney were removed for histological studies and were fixed immediately in 10% neutral buffered formalin (13, 22, 32).

#### 2.4.1 Enzyme assay

Acetylcholinesterase (AChE) activity was measured by the colorimetric method used by Kašuba et al. (36) with little modifications, using acetylthiocholine iodide (ATChI) as the enzymatic substrate. The tissue homogenates were prepared in 0.1 M phosphate buffer (pH 7.4) and centrifuged at 10,000 × g for 10 min at 4 °C. An aliquot (50–100 μL) of the resulting supernatant was added to the reaction mixture containing 0.1 M phosphate buffer (pH 8.0), 1 mM 5,5′-dithiobis-(2-nitrobenzoic acid) (DTNB), and 1 mM ATChI in a final reaction volume of 3 mL. The formation of 5-thio-2-nitrobenzoate (TNB) was monitored spectrophotometrically at 412 nm for 3–5 min at room temperature. Enzyme activity was calculated from the linear rate of absorbance change using the molar extinction coefficient (*ε* = 13,600 M^−1^ cm^−1^) and expressed as U/mg protein, where one unit (U) represents the hydrolysis of 1 nmol of substrate per minute under assay conditions. The activity of ALP in the serum samples was determined using the Erba ALP kit, according to the kit instructions. The serum samples were set up in the reaction wells, and substrate solution containing p-nitrophenyl phosphate, pNPP, was added. The substrate, pNPP, is hydrolyzed by the ALP enzyme to produce p-nitrophenol, which produces a yellow color detectable with a wavelength of 405 nm using a spectrophotometer after the reaction mixture is allowed to reach room temperature for the recommended time, and the reaction is then arrested using the stop solution supplied with the kit. Activity of ALP was determined based on absorbance measurements utilizing a standard curve that was obtained from a calibrator solution provided in the kit. It was measured in U/L. One unit of ALP was defined as the amount that carries out 1 μmol p-nitrophenyl phosphate per minute. All samples for ALP activity measurements were measured in duplicates. Absorbance was read using a microplate reader (37). The level of different hepatic enzymes in blood was determined from the serum collected from different treatment groups (33, 36, 38, 39).

#### 2.4.2 Analysis of histopathology

Preserved organs were processed for histopathological studies as described by Bancroft and Gamble (51). Tissues were treated with 10% buffered formalin for fixation and cut into 5 mm thick pieces and placed in water overnight to remove all residue of fixative. Slices of tissues were then dehydrated gradually in 70, 85, 95%, Absolute alcohol-I and Absolute alcohol-2 for 8, 4, 4, 2, and 2 h, respectively. Dehydrating agent was eliminated by cleaning with Xylene + Absolute Alcohol (50 + 50), Xylene-1, and Xylene-2 for 30, 15, and 15 min, respectively. Infiltration was carried out in liquid paraffin at 59–60 °C for 2 h. After embedding and staining by using H and E stain adopting protocols, a drop of DPX was put on the cover slip, and it was placed on the stained section, avoiding bubble formation. Afterward 3–4 μm thick section will be studied under a light microscope at 4, 20, 40, and 100 magnifications. Slides of all the treated groups were studied, and photographs were taken (21, 22, 32).

### 2.5 Analysis of hematological parameters

All blood samples were collected at every 7, 14, 21, and 28 days in serum vials containing EDTA solution, and serum was separated by placing the samples into a centrifugal machine by centrifugation at 3000 rpm for 10 min. As already described, the rats were sacrificed at every 7, 14, and 28 days, respectively. Detailed postmortem examinations were conducted by opening up the belly of the rats, gross lesions, fatty lesions, organ color, and internal hemorrhages were noted, the liver and kidney were fixed in 10% formalin for histology, and in phosphate buffer saline for enzymatic assay.

The blood serum from collected blood samples was used to determine the level of hepatic enzymes (bilirubin, S. G. O. T, and S. G. P. T), A. phosphatase (ALP) by using Armstrong method, and kidney enzymes, uric acid, urea (Diacetyl Monoxide Method), and creatinine (Modified Jaffer’s Method) (21, 22).

The blood parameters were analyzed by using a hematology auto analyzer CELL-DYN for the analysis of red blood cells (RBC, 10^6^ cells/mm^3^) count, white blood cells (WBC, 10^3^ cells/mm^3^) counts, hemoglobin (Hb, g/dL), platelets (total thrombocytes count, 10^4^ cells/mm^3^), differential leucocytes count (DLC), including numbers of Lymphocytes, neutrophils, monocytes, Eosinophil per 100 cells, total leukocytes count (TLC, 10^3^ cells/mm^3^), HCT (%), MCV, MCH, and MCHC (%).

### 2.6 Genotoxicity assessment through comet assay

To analyze the DNA damage due to different treatments, the comet assay was performed following the protocol described by Attaullah et al. (30) and Khalid et al. (40).

### 2.7 Statistical analysis

Results were statistically analyzed through ANOVA. The results are presented as the mean for each group. Differences among groups were analyzed using a one-way analysis of variance (ANOVA) test. Means were separated by using Tukey’s HSD test at a significance level of 0.05. A *p*-value < 0.05 was considered statistically significant (28, 33, 41, 42).

## 3 Results

Phytochemical constituents of both weed plants were characterized using biochemical assay and through Fourier Transform Infrared Spectroscopy (FTIR) by our laboratory (19). Both *Achyranthes aspera and Chenopodium murale* showed the presence of Flavonoids, Saponins, Tannins, Steroids, Cardiac glycosides, and Alkaloids. However, *Achyranthes aspera* also showed anthraquinones and terpenoids. FTIR spectrum of *Achyranthes aspera* revealed peaks corresponding to –OH group (phenolic group), alkyl C-H group, C=H group, C-O stretching, and CH bending for aromatic ring. Overall, FTIR results revealed the presence of phenolic compounds in the petroleum ether extract of *A. aspera*. The FTIR spectrum for *Chenopodium murale* revealed the presence of stretching peaks corresponding to O-H, -CH_2_, C=O, and C-H groups. The bands were found as an indication of phenolic compounds having carboxylic acids present in the organic extracts of *Chenopodium murale* (19, 20).

### 3.1 Enzyme activity

#### 3.1.1 Acetylcholine esterase (AChE)

The activity of AChE was measured in cypermethrin and plant extracts; *C. murale* and *A. aspera*, treated groups, and compared with the control group after 7, 14, 21, and 28 days, respectively. AChE activity was decreased with an increase in concentrations and exposure time, as shown in Table 1. Cypermethrin showed significantly low activity of AChE, i.e., 0.217, 0.203, and 0.172 as compared to control at the concentrations 100, 150, and 250 ppm, respectively, after 28 days following the activity of AChE; 0.255, 0.243, and 0.235 after 7 days; 0.248, 0.240 and 0.213 after 14 days; and 0.232, 0.228 and 0.187 after 21 days at 100, 150 and 250 ppm concentrations of cypermethrin, respectively. AChE activity was significantly (*p* < 0.05) decreased with increasing exposure time and concentrations of cypermethrin, while the treatment with plant extracts showed a non-significant (*p* > 0.05) difference in the activity of AChE as compared to the control group (Table 1).

**Table 1:** The effect of cypermethrin and ethanolic plant extracts at various doses and post treatment intervals (7, 14, 21, and 28 days) on enzyme activity in albino rat.

Treatment	Days	AChE				AlkP			
Conc./ppm	–	Control	100 ppm	150 ppm	250 ppm	Control	100 ppm	150 ppm	250 ppm
Cyper	7 days	0.278 ± 0.007a	0.255 ± 0.002c	0.243 ± 0.01b	0.235 ± 0.001b	253 ± 3.56a	276 ± 4.32b	280 ± 1.22c	287 ± 0.68c
		(*F* = 12.75;d.f. = 3; *p* = 0.00)				(*F* = 13.26; d.f. = 3; *p* = 0.000)			
	14 days	0.278 ± 0.007a	0.248 ± 0.003b	0.240 ± 0.005b	0.213 ± 0.002c	253 ± 3.56a	278 ± 3.12ab	291 ± 2.16c	301 ± 1.27c
		(*F* = 11.55;d.f. = 3; p = 0.00)				(*F* = 16.20; d.f. = 3; p = 0.000)			
	21 days	0.278 ± 0.007a	0.232 ± 0.005b	0.228 ± 0.007b	0.187 ± 0.003c	253 ± 3.56a	285 ± 6.31b	298 ± 5.77b	317 ± 2.30d
		(*F* = 10.25;d.f. = 3; p = 0.00)				(*F* = 17.73; d.f. = 3; p = 0.000)			
	28 days	0.278 ± 0.007a	0.217 ± 0.003b	0.203 ± 0.002c	0.172 ± 0.009d	253 ± 3.56a	291 ± 4.71b	307 ± 2.63c	321 ± 3.82d
		(*F* = 9.25;d.f. = 3; P = 0.00)				(*F* = 18.19; d.f. = 3; P = 0.000)			
P1	7 days	0.278 ± 0.007a	0.278 ± 0.003a	0.278 ± 0.009a	0.275 ± 0.005a	253 ± 3.56a	247 ± 6.17a	253 ± 1.96a	256 ± 4.03a
		(*F* = 13.61;d.f. = 3; *p* = 0.02)				(*F* = 12.77; d.f. = 3; *p* = 0.054)			
	14 days	0.278 ± 0.007a	0.279 ± 0.004a	0.276 ± 0.003a	0.276 ± 0.002a	253 ± 3.56a	245 ± 5.77a	251 ± 2.57a	255 ± 2.88a
		(*F* = 12.81;d.f. = 3; *p* = 0.012)				(*F* = 11.31; d.f. = 3; *p* = 0.033)			
	21 days	0.278 ± 0.007a	0.280 ± 0.002a	0.278 ± 0.005a	0.277 ± 0.004a	253 ± 3.56a	250 ± 2.72a	253 ± 0.57a	254 ± 2.13a
		(*F* = 10.63;d.f. = 3; *p* = 0.045)				(*F* = 10.58; d.f. = 3; *p* = 0.064)			
	28 days	0.278 ± 0.007a	0.279 ± 0.06a	0.275 ± 0.005a	0.275 ± 0.002a	253 ± 3.56d	252 ± 3.46a	254 ± 1.73a	256 ± 2.88a
		(*F* = 11.34;d.f. = 3; *p* = 0.032)				(*F* = 10.33; d.f. = 3; *p* = 0.080)			
P2	7 days	0.278 ± 0.007a	0.276 ± 0.004a	0.275 ± 0.002a	0.275 ± 0.003a	253 ± 3.56a	249 ± 4.76a	252 ± 2.28a	255 ± 2.79a
		(*F* = 10.56;d.f. = 3; *p* = 0.048)				(*F* = 10.60; d.f. = 3; *p* = 0.063)			
	14 days	0.278 ± 0.007a	0.278 ± 0.003a	0.277 ± 0.005a	0.274 ± 0.003a	253 ± 3.56a	253 ± 3.45a	253 ± 4.84a	258 ± 5.14a
		(*F* = 11.13;d.f. = 3; *p* = 0.039)				(*F* = 10.40; d.f. = 3; *p* = 0.075)			
	21 days	0.278 ± 0.007a	0.278 ± 0.003a	0.276 ± 0.001a	0.276 ± 0.007a	253 ± 3.56a	256 ± 3.72a	256 ± 4.27a	257 ± 4.65a
		(*F* = 11.41;d.f. = 3; *p* = 0.030)				(F = 10.25; d.f. = 3; *p* = 0.085)			
	28 days	0.278 ± 0.007a	0.276 ± 0.005a	0.275 ± 0.007a	0.273 ± 0.005a	253 ± 3.56a	258 ± 3.46a	257 ± 4.96a	258 ± 5.02 a
		(*F* = 12.00;d.f. = 3; *p* = 0.019)				(*F* = 10.42; d.f. = 3; *p* = 0.73)			

Cyper, Cypermethrin; P1, *C. murale*; P2, *A. asper*a; *p* < 0.05, Significant; same letters, non-significant.

#### 3.1.2 Activity of AlkP

Similarly, the activity of AlkP was measured in cypermethrin and both the plants extracts, *C. murale* and *A. aspera,* treated groups, and compared with the control group after 7, 14, 21, and 28 days. Results showed that AlkP activity was increased with increasing concentrations and exposure time (Table 1). Cypermethrin showed the significant higher activity of AlkP (291, 307, and 321) as compared to control at the concentrations 100, 150, and 250 ppm, respectively, after 28 days following the activity of AlkP (276, 280, and 287), (278, 291, and 301) and (285, 298, and 317) at (100, 150, and 250 ppm) concentrations of cypermethrin, respectively, after 7, 14, and 21 days, respectively. AlkP activity was significantly (*p* < 0.05) increased with increasing exposure time and concentrations of cypermethrin, while the treatment with plant extracts showed a non-significant (*p* > 0.05) difference in activity of AlkP as compared to the control group (Table 1).

### 3.2 Histopathology of liver tissue

**Figure 1:**
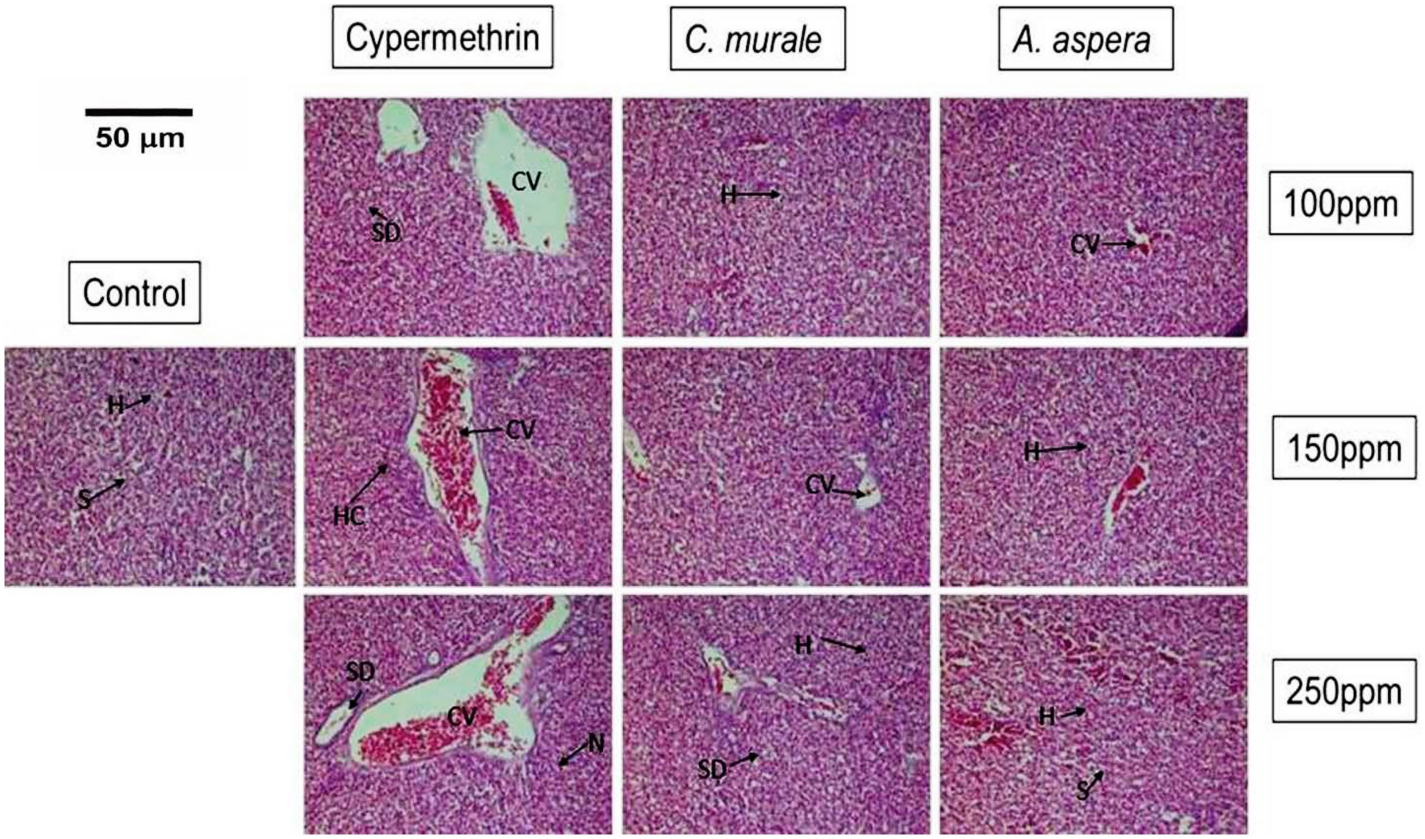
Histological comparison of liver tissues: Control, cypermethrin, and plant extracts; *C. murale* and *A. aspera* groups at different doses (28 days). Control group: Normal hepatic architecture with undamaged hepatocytes (H), central veins (CV), and regular sinusoids (S). Cypermethrin-treated group: Hepatocyte necrosis, congestion, cytoplasmic vacuolation (V), and sinusoidal dilatation (SD) in a dose-dependent liver injury. *C. murale* and *A. aspera*: Normal hepatic cords, decreased vascular congestion, and nearly normal architecture. These results show that cypermethrin induced hepatotoxicity, while both plant extracts had no changes in liver tissue.

Histological comparison was performed between liver tissues of cypermethrin and plant extracts; *C. murale* and *A. aspera* groups with respect to control at different doses (100, 150, and 250 ppm) and different time periods (7, 14, 21, and 28 days) by light microscopy. The control group showed regular and compact structure with well-organized hepatic cells and central veins. No toxic effect of *C. murale* and *A. aspera* plant extracts was observed on the liver structure of albino rats at different doses and different time periods. Plant extracts treated group indicated about the same structure of liver as the control group, while cypermethrin caused severe damage to liver tissue and showed different histopathological alterations at different doses and times. The cypermethrin-treated albino rats group showed different histopathological alterations at different doses and times. The treatment group of cypermethrin for 7 days showed moderate enlargement of sinusoids, vacuole formations in hepatocytes, and congestion in the central vein; the exposure of cypermethrin for 14 days caused enlargement in the sinusoidal space and hemorrhages in liver tissue. However, most of the intrahepatic blood vessels, especially the central veins, were dilated and congested at 21 days (data not shown). The treatment of cypermethrin for 28 days showed that some areas of liver tissue appeared with necrosis infiltrated with mononuclear cells. In addition, the hepatocytes lost their normal architecture and vacuolization, and pyknotic nuclei appeared in the cytoplasm. Overall, the liver section of the cypermethrin treatment group showed granulomas (macrophage collection), indicating an immune response to inflammation (granulomatous inflammation) and infection in the portal region or necrosis of liver tissues, dilation or severe congestion of the intrahepatic blood vessels, especially the central veins, and hemorrhage in hepatic tissue (Figure 1 and Table 2). Due to cypermethrin exposure, three rats died between 16 and 28 days. The most serious damage to liver tissues was observed at the highest dose (250 ppm) of cypermethrin at 28 days. However, both the plants, *Chenopodium murale* and *Achyranthes aspera,* showed about the same liver structure as the control group (Figure 1 and Table 2).

**Table 2:** Quantitative histopathological scoring of liver tissues.

Sr. #	Parameter	Cypermethrin	*C. murale*	*A. aspera*
1	Hepatocytes (H)	**+++**	**+**	**+**
2	Central veins (CV)	**+++**	**+**	**+**
3	Sinusoids (S)	**+++**	**++**	**++**
4	Congestion	**+++**	**+**	**+**
5	Cytoplasmic vacuolation (V)	**+++**	**+**	**+**
6	Sinusoidal dilatation (SD)	**+++**	**+**	**+**

The damage/change has been normalized to control.

### 3.3 Hepatic enzymes parameters

Level of liver enzymes in serum, i.e., S. bilirubin, SGOT, SGPT, and A. phosphatase, were determined after 7, 14, 21, and 28 days of exposure of albino rats to cypermethrin and *C. murale* and *A. aspera* plant extracts. It was revealed that serum bilirubin was significantly increased after 7 days with cypermethrin as compared to control; it was 2.3, 2.4, and 2.9 mg/dL at 100, 150, and 250 ppm concentrations of cypermethrin, respectively. S. bilirubin was increased with exposure time and concentrations of cypermethrin. The highest level of S. bilirubin was observed after 28 days in cypermethrin treated group; 3, 3.2, and 3.59 mg/dL at 100, 150 and 250 ppm concentrations of cypermethrin, respectively. Similarly S.G.O.T was found highest after 28 days in cypermethrin treated group; 68, 80, 89 u/L following 65, 66, and 78 u/L after 14 days, and 67, 78, and 84 u/L after 21 days at 100, 150 and 250 ppm concentrations of cypermethrin, respectively (Table 3). Hence, S. bilirubin level was increased significantly (*p* < 0.05) as compared to control at all concentrations of cypermethrin after 7, 14, 21, and 28 days exposure of albino rats to cypermethrin; however, the S. bilirubin level was found non-significant (*p* > 0.05) in control group and *C. murale* and *A. aspera* plant extracts at all concentrations and exposure time of 7, 14, 21, and 28 days (Table 3). After 7 days, S.G.O.T level was 60, 62, and 70 u/L at 100, 150, and 250 ppm concentrations of cypermethrin, respectively. The highest level of S.G.P.T was 61, 68, and 76 u/L at 100, 150, and 250 ppm concentrations of cypermethrin, respectively, after 28 days following 7, 14, and 21 days with level of S.G.P.T 50, 52, and 55 u/L; 54, 55, and 59 u/L; and 56, 58, and 63u/L, respectively, at 100, 150, and 250 ppm concentrations, respectively (Table 3). The highest level of A. phosphatase was 360, 388, and 397 ul at 100, 150, and 250 ppm concentrations of cypermethrin, respectively, after 28 days, following 7, 14, and 21 days with 350, 354, and 359 u/L; 353, 356, and 364u/L; and 355, 380, and 390 u/L, respectively, at concentrations of 100, 150, and 250 ppm, respectively. The level of all studied enzymes of the liver in the blood was significantly (>0.05) higher as compared to control at all exposure times and concentrations of cypermethrin, while the treatment of plant extracts showed a non-significant difference in the level of enzymes as compared to control (Table 3).

**Table 3:** The effect of cypermethrin and ethanolic plant extracts at various doses and post treatment intervals (7, 14, 21, and 28 days) on the level of some hepatic enzymes in the blood serum of Albino rat.

7th day of treatment											
Parameters	Ref. value	Control group	Cypermethrin			*Chenopodium murale*			*Achyranthes aspera*		
			100 ppm	150 Ppm	250 ppm	100 ppm	150 ppm	250 ppm	100 Ppm	150 ppm	250 ppm
*S. bilirubin* (mg/dL)	0.2–1.2	0.34 ± 0.005a	2.3 ± 0.01b	2.4 ± 0.01b	2.9 ± 0.02c	0.36 ± 0.05a	0.38 ± 0.03a	0.40 ± 0.05a	0.32 ± 0.01a	0.38 ± 0.05a	0.32 ± 0.03a
S. G. O. T (u/L)	10–35	15 ± 0.57a	60 ± 1.05c	62 ± 1.12c	70 ± 1.03c	18 ± 2.77a	20 ± 5.04a	20.5 ± 4.63a	16 ± 1.89a	18 ± 1.22a	17 ± 0.63a
S. G. P. T (u/L)	9–34	17 ± 0.57a	50 ± 2.01b	52 ± 1.67c	55 ± 0.26c	16.5 ± 2.57a	19 ± 2.73a	19 ± 1.92a	18 ± 1.57a	17 ± 1.05a	20 ± 2.58 a
A. phosphatase (u/L)	65–306	268 ± 2.76a	350 ± 0.01b	354 ± 0.01b	359 ± 0.01bc	268 ± 1.46a	272 ± 3.02a	273 ± 2.84a	270 ± 0.59a	272 ± 1.07a	273 ± 1.99a
14th day of treatment											
S. bilirubin (mg/dL)	0.2–1.2	0.40 ± 0.05a	2.5 ± 0.01b	2.6 ± 0.02bc	2.96 ± 0.03c	0.42 ± 0.01a	0.45 ± 0.03a	0.47 ± 0.05a	0.42 ± 0.02a	0.43 ± 0.01a	0.45 ± 0.02a
S. G. O. T (u/L)	10–35	30 ± 0.57a	65 ± 0.63b	66 ± 0.79b	78 ± 0.47c	29 ± 0.28a	28 ± 0.57a	30 ± 0.73a	30 ± 0.53a	31 ± 0.05a	33 ± 1.47a
S. G. P. T (u/L)	9–34	25 ± 0.57a	54 ± 0.03b	55 ± 0.72b	59 ± 0.15bc	23 ± 2.05a	26 ± 1.07a	27 ± 2.03a	24 ± 1.15a	26 ± 1.05a	28 ± 2.79a
A.phosphatase (u/L)	65–306	270 ± 3.21a	353 ± 3.34b	356 ± 2.78b	364 ± 3.59c	272 ± 1.58a	272 ± 0.52a	274 ± 1.33a	271 ± 0.63a	273 ± 1.67a	275 ± 3.95a
21st day of treatment											
*S. bilirubin* (mg/dL)	0.2–1.2	0.50 ± 0.05a	2.7 ± 0.05b	2.8 ± 0.01b	3.0 ± 0.03c	0.48 ± 0.14a	0.52 ± 0.01a	0.53 ± 0.02a	0.50 ± 0.01a	0.51 ± 0.03a	0.55 ± 0.04a
S. G. O. T (u/L)	10–35	30 ± 0.57a	67 ± 0.01b	78 ± 0.05c	84 ± 0.01c	30.5 ± 0.59a	32 ± 1.75a	33 ± 2.80a	31 ± 0.78a	33 ± 2.17a	33.5 ± 1.56a
S. G. P. T (u/L)	9–34	38 ± 0.05a	56 ± 0.78b	58 ± 1.02b	63 ± 0.89c	37 ± 0.57a	39 ± 0.95a	41 ± 0.86a	40 ± 1.68a	39.5 ± 0.93a	39.9 ± 1.24a
A. phosphatase (u/L)	65–306	275 ± 3.81a	355 ± 3.57b	380 ± 2.73c	390 ± 5.38d	277 ± 2.01a	277 ± 1.81a	279 ± 1.57a	276 ± 0.63a	278 ± 1.84a	280 ± 2.53a
28th day of treatment											
S. bilirubin (mg/dL)	0.2–1.2	0.52 ± 0.04a	3.0 ± 0.05b	3.2 ± 0.15c	3.5 ± 0.68c	0.51 ± 0.02a	0.53 ± 0.02a	0.52 ± 0.03a	0.52 ± 0.02a	0.53 ± 0.01a	0.54 ± 0.03a
S. G. O. T (u/L)	10–35	31 ± 0.57a	68 ± 0.09b	80 ± 0.06c	89 ± 1.72 cd	31 ± 0.64a	33 ± 1.03a	33 ± 1.38a	32 ± 1.05a	33.5 ± 1.57a	34 ± 2.07a
S. G. P. T (u/L)	9–34	37 ± 1.53a	61 ± 1.21b	68 ± 1.76c	76 ± 2.25c	38 ± 1.03a	39 ± 0.63a	41 ± 0.68a	41 ± 1.16a	40 ± 0.68a	41 ± 1.09a
A. phosphatase (u/L)	65–306	290 ± 4.32a	360 ± 1.67b	388 ± 1.86c	397 ± 1.05 cd	291 ± 0.54a	290 ± 0.69a	293 ± 3.22a	292 ± 2.07a	292 ± 1.97a	293 ± 2.60a

A. phosphatsase, acid phosphatase; *p* < 0.05, Significant; same letters, non-significant.

### 3.4 Histopathological findings of the kidney

**Figure 2:**
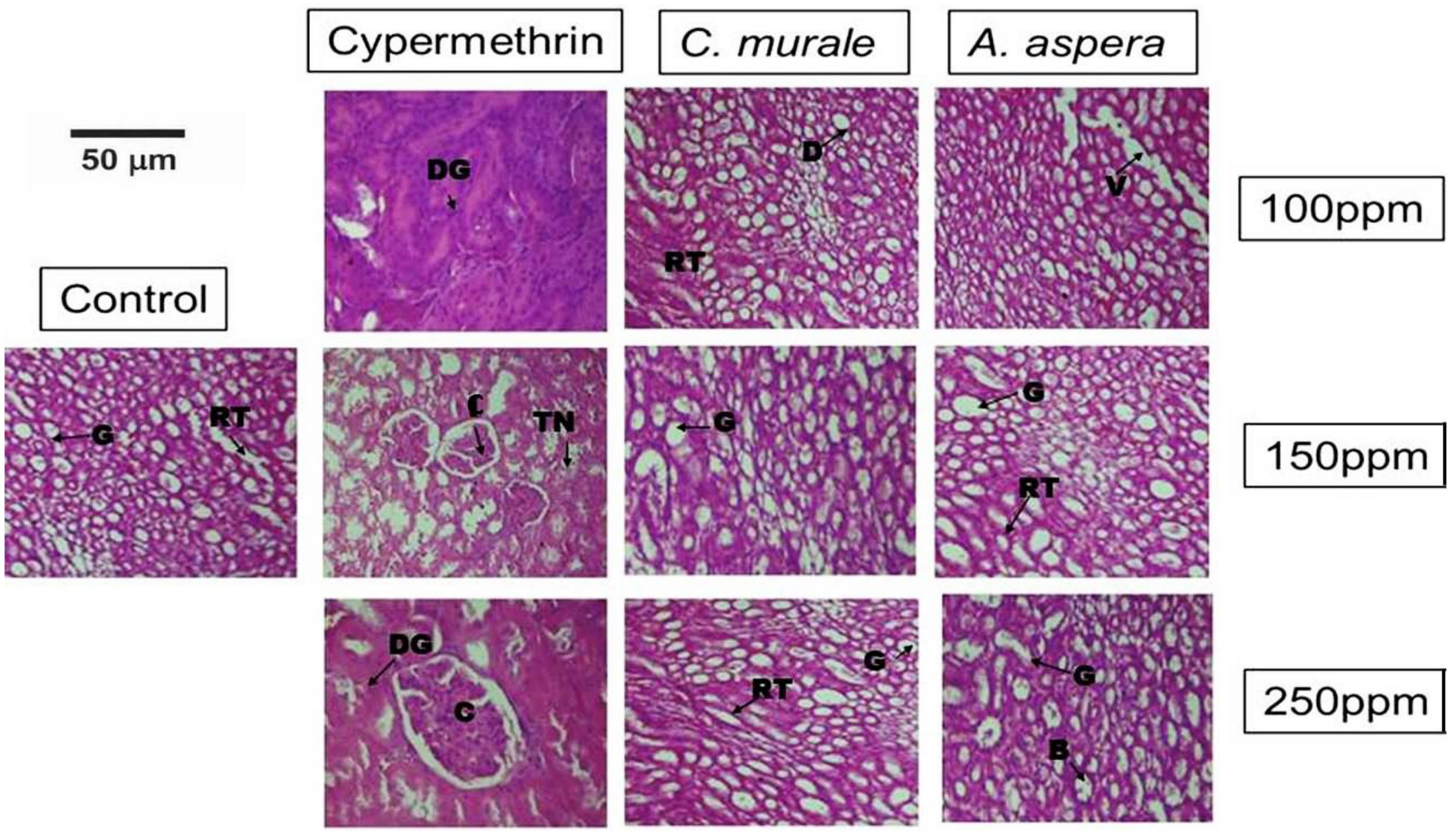
Histological comparison of kidney tissues: Control, cypermethrin, and plant extracts; *C. murale* and *A. aspera* groups at different doses (28 days). Control group: Kidney with intact glomeruli, a small Bowman’s capsule (B), and neatly aligned renal tubules (RT). Control shows normal renal histoarchitecture. Cypermethrin-treated group: Glomerular degeneration (DG) and vascular congestion (C), tubular necrosis (TN), and epithelial desquamation are among the progressive nephrotoxic changes. *C. murale* and *A. aspera*: Renal tubules (RT), glomerulus (G), Bowman’s capsule (B), vessel (V), and degeneration (D). Plant extracts show almost normal glomeruli with little tubular dilatation (D).

Histopathological analysis of kidney tissue at different concentrations of ethanolic extracts of *C. murale* and *A. aspera*, and cypermethrin was observed in male albino rats. Cypermethrin showed significant changes in kidney tissues at all concentrations and exposure times as compared to the control group. The plant extracts did not show any significant changes in tissue even at the highest concentration (250 ppm) as compared to the control group. The transverse structure of the kidney cells in the control group showed ordinary arrangements of Bowman’s capsule, urinary pulp, glomerulus, proximal and distal convoluted tubules, and podocytes. However, oral doses of cypermethrin produced significant changes in the histology of kidney tissue, especially at high dosage 250 ppm after 28 days. Cypermethrin-treated group kidney structure showed necrosis of renal tubules, fibrosis, and swelling in Bowman’s capsule in the treatment period of 28 days. *C. murale and A. aspera* had no adverse effect on the kidney at the time and dose range used in the present study. There was no significant modification in the kidney, nor did the plant extracts cause any alteration in the micro-arrangement of the kidney (Figure 2 and Table 4).

**Table 4:** Quantitative histopathological scoring of kidney tissues.

Sr. #	Parameter	Cypermethrin	*C. murale*	*A. aspera*
1	Glomerulus (G)	**+++**	**++**	**++**
2	Bowman’s capsule (B)	**+++**	**+**	**+**
3	Vascular congestion (C)	**+++**	**+**	**++**
4	Degeneration (D)	**+++**	**+**	**+**
5	Tubular necrosis (TN)	**+++**	**+**	**+**
6	Renal tubules (RT)	**+++**	**++**	**+**

The damage/change has been normalized to control.

### 3.5 Kidney enzyme parameters

Histopathological results of the cypermethrin and both the extracts treated rats are shown in Table 3. Non-significant results were found after treatment with plant extracts compared to the control group. Enzyme histology of kidney tissue revealed the value of the control group as 15 mg/dL, which is non-significant and safer against a reference value of S. urea 10–50 mg/dL. A significant increase was observed in the cypermethrin-treated group, whereas non-significant results were found with both plant extracts. Creatinine has values between 0.6 and 1.2 when compared with the control group, which showed a 0.43 mg/dL value. Uric acid was found between 3.4 mg/dL and the comparison with the control group, 3.6 mg/dL, showed a highly non-significant difference. Creatinine, urea, and uric acid of both plant extracts have non-significant results when compared with the control group. No sharp difference in hematological parameters was observed (Table 5).

**Table 5:** Comparison of the means of the effect of ethanolic plant extract and cypermethrin at various doses and post treatment intervals on kidney enzymes of albino rat at 7, 14, 21, and 28 days.

Parameters	Reference value	Control group	Cypermethrin			*C. murale*			*A. aspera*		
			100 ppm	150 ppm	250 ppm	100 ppm	150 ppm	250 ppm	100 ppm	150 ppm	250 ppm
7 days											
Urea (mg/dL)	10–50	15 ± 0.5c	51 ± 0.5a	52 ± 0.5a	53 ± 0.5a	17 ± 0.0c	20 ± 0.0c	22 ± 0.0bc	22 ± 0.5bc	24 ± 0.0bc	26 ± 0.0bc
*S. creatinine* (mg/dL)	0.6–1.2	0.37 ± 0.5b	0.6 ± 0.0a	0.7 ± 0.0a	0.8 ± 0.2a	0.39 ± 0.8b	0.40 ± 0.5b	0.42 ± 0.1a	0.44 ± 0.5b	0.46 ± 0.5b	0.48 ± 0.5b
*S. uric* acid (mg/dL)	3.4–7.1	4.0 ± 0.5b	7.9 ± 0.3a	8.1 ± 0.5a	8.2 ± 0.5a	4.2 ± 0.5b	4.6 ± 0.5b	4.8 ± 0.5b	4.6 ± 0.5b	4.8 ± 0.5b	4.9 ± 0.5b
14 days											
Urea (mg/dL)	10–50	15 ± 0.5d	53 ± 0.5a	53 ± 0.5a	57 ± 0.5ab	21 ± 0.0d	22 ± 0.0d	24 ± 0.0 cd	24 ± 0.5 cds	26 ± 0.0c	29 ± 0.0c
*S. creatinine* (mg/dL)	0.6–1.2	0.37 ± 0.5b	0.7 ± 0.0a	0.9 ± 0.0a	0.8 ± 0.2a	0.42 ± 0.2b	0.44 ± 0.5b	0.46 ± 0.1b	0.44 ± 0.5b	0.46 ± 0.5b	0.48 ± 0.5b
*S. uric* acid (mg/dL)	3.4–7.1	4.0 ± 0.5b	7.9 ± 0.3a	8.5 ± 0.5a	8.7 ± 0.5a	4.8 ± 0.5b	5.0 ± 0.5b	5.2 ± 0.5b	5.4 ± 0.5b	5.6 ± 0.5b	5.8 ± 0.5b
21 days											
Urea (mg/dL)	10–50	15 ± 0.5c	52 ± 0.5a	53 ± 0.5a	54 ± 0.5a	22 ± 0.0c	24 ± 0.0c	27 ± 0.00bc	27 ± 0.05bc	30 ± 0.00b	35 ± 0.00b
*S. creatinine* (mg/dL)	0.6–1.2	0.37 ± 0.5b	0.9 ± 0.0a	0.8 ± 0.0a	0.9 ± 0.2a	0.50 ± 0.2b	0.52 ± 0.5b	0.54 ± 0.1b	0.56 ± 0.5b	0.57 ± 0.5b	0.59 ± 0.5b
*S. uric* acid (mg/dL)	3.4–7.1	4.0 ± 0.5b	7.9 ± 0.3a	8.9 ± 0.5a	8.9 ± 0.5a	6.0 ± 0.5b	6.2 ± 0.5b	6.4 ± 0.5b	6.6 ± 0.5b	6.8 ± 0.5b	6.9 ± 0.5b
28 days											
Urea (mg/dL)	10–50	15 ± 0.5c	51 ± 0.5a	52 ± 0.5a	53 ± 0.5a	27 ± 0.0bc	29 ± 0.00bc	30 ± 0.00bc	32 ± 0.5bc	34 ± 0.00b	36 ± 0.00b
*S. creatinine* (mg/dL)	0.6–1.2	0.37 ± 0.5b	0.6 ± 0.0a	0.7 ± 0.0a	0.8 ± 0.2a	0.50 ± 0.8b	0.52 ± 0.5b	0.54 ± 0.1b	0.46 ± 0.5b	0.58 ± 0.5b	0.60 ± 0.5b
*S. uric* acid (mg/dL)	3.4–7.1	4.0 ± 0.5b	7.9 ± 0.3a	8.1 ± 0.5a	8.2 ± 0.5a	6.2 ± 0.5b	6.4 ± 0.5b	6.6 ± 0.5b	7.0 ± 0.0b	7.2 ± 0.5b	7.4 ± 0.5b

*p* < 0.05, Significant; same letters, non-significant.

### 3.6 Hematological parameters

The blood parameters of treated rats were analyzed which revealed significant changes in blood parameters (Hb, TLC, RBCs, HCT, MCH, MCHC, Platelets, Neutrophils, Lymphocytes, Monocytes, and Eosinophils) against administration of different doses (100, 150, and 250 ppm) of cypermethrin at 28 days while both plant extracts, *C. murale* and *A. aspera*, did not show significant changes in these parameters as compared to control group. However, *C. murale* and *A. aspera* showed an increase in MCV, percentage of neutrophils, and lymphocytes at 150 and 250 ppm dose concentrations. *A. aspera* showed a significant decrease in platelets as compared to control at 250 ppm, but this decrease was slightly less than that of cypermethrin at all doses. Cypermethrin showed a significant decrease in platelets and Hb but an increase in TLC and RBCs. HCT, MCV, MCH, MCHC, Neutrophils, Lymphocytes, Monocytes, and Eosinophils; while both plant extracts did not showed significant increase in these parameters except MCV, neutrophils, and lymphocytes (Table 6 and Supplementary Tables 1–3).

**Table 6:** Means comparison of the data pertaining to the effect of ethanolic plant extract at various doses and post treatment intervals on blood parameters of the albino rat at 28 days.

Parameters	Reference value	Control group	Cypermethrin			*C. murale*			*A. aspera*		
			100 ppm	150 ppm	250 ppm	100 ppm	150 ppm	250 ppm	100 ppm	150 ppm	250 ppm
Haemoglobin (g/dl)	14–18	14.0 ± 0.5a	11.20 ± 0.5b	8.50 ± 0.1c	9.33 ± 0.5c	14.20 ± 0.5a	14.53 ± 0.5a	14.20 ± 0.3a	14.70 ± 0.5 a	14.53 ± 0.5a	14.20 ± 0.5a
T. L. C (X10^9^/L)	4.0–11.0	5.8 ± 0.5	10.16 ± 0.5b	13.5 ± 0.2bc	15.7 ± 0.1c	6.0 ± 0.5 a	5.5 ± 0.5a	5.3 ± 0.5a	5.8 ± 0.5a	5.5 ± 0.5a	5.3 ± 0.5c
R. B. C(x10^12^/L)	4.6–6.0	7.20 ± 0.5a	7.72 ± 0.2b	7.50 ± 0.5b	8.33 ± 0.5c	7.94 ± 0.5ab	7.45 ± 0.1a	7.33 ± 0.5a	7.6 ± 0.5ab	7.4 ± 0.1a	7.3 ± 0.5b
HCT (%)	40–54	37.9 ± 0.5a	72 ± 0.5b	78 ± 0.1bc	80 ± 0.2c	40.80 ± 0.5a	39.00 ± 0.5a	38.50 ± 0.5a	41.5 ± 0.5ab	40.0 ± 0.5a	39.9 ± 0.5a
MCV (fi)	80–93	80.1 ± 0.5a	98 ± 0.1b	104 ± 0.2c	105 ± 0.1c	83.5 ± 0.5a	86.5 ± 0.5a	88.0 ± 0.5a	93 ± 0.5ab	94 ± 0.5ab	95 ± 0.5ab
MCH(pg)	26–32	25 ± 0.5a	55 ± 0.5b	58 ± 0.3b	60 ± 0.2bc	24.20 ± 0.5a	25.00 ± 0.5a	25.90 ± 0.5a	29.4 ± 0.5a	29.9 ± 0.5a	31 ± 0.5ab
MCHC (%)	32–36	36 ± 0.5a	43 ± 0.1b	46 ± 0.3b	48 ± 0.5bc	32.40 ± 0.5a	32.50 ± 0.5a	32.00 ± 0.5a	34 ± 0.5a	33 ± 0.5a	33.2 ± 0.5a
Platelets (x10^9^/L)	150–400	850 ± 0.5a	500 ± 0.5b	550 ± 0.5b	600 ± 0.2c	825 ± 0.5a	835 ± 0.5a	850 ± 0.5a	800 ± 0.5a	790 ± 0.5ab	750 ± 0.5ab
Neutrophils %	35–65	38 ± 0.5a	74 ± 0.5b	77 ± 0.2b	78 ± 0.1b	48 ± 0.5a	52 ± 0.5a	55 ± 0.5a	63 ± 0.5a	67 ± 0.5b	69 ± 0.5b
Lymphocytes %	23–53	32 ± 0.5a	60 ± 0.5b	62 ± 0.1c	64 ± 0.2c	45.0 ± 0.5a	47.0 ± 0.5a	48.0 ± 0.5a	50 ± 0.5a	58 ± 0.5b	62 ± 0.5b
Monocytes %	2–11	2 ± 0.5a	19 ± 0.2b	20 ± 0.3bc	28 ± 0.5c	3.4 ± 0.5a	3.5 ± 0.5a	3.7 ± 0.5a	3.4 ± 0.5a	3.5 ± 0.5a	3.7 ± 0.5a
Eosinophils %	1–4	2 ± 0.5a	12 ± 0.2b	13 ± 0.5bc	18 ± 0.5c	3.3 ± 0.5a	3.6 ± 0.5a	3.9 ± 0.5a	3.7 ± 0.5a	3.9 ± 0.5a	3.9 ± 0.5a

*p* < 0.05, Significant; same letters, non-significant.

### 3.7 Genotoxic effects in albino rats due to exposure to different concentrations of cypermethrin and plant extracts

**Figure 3:**
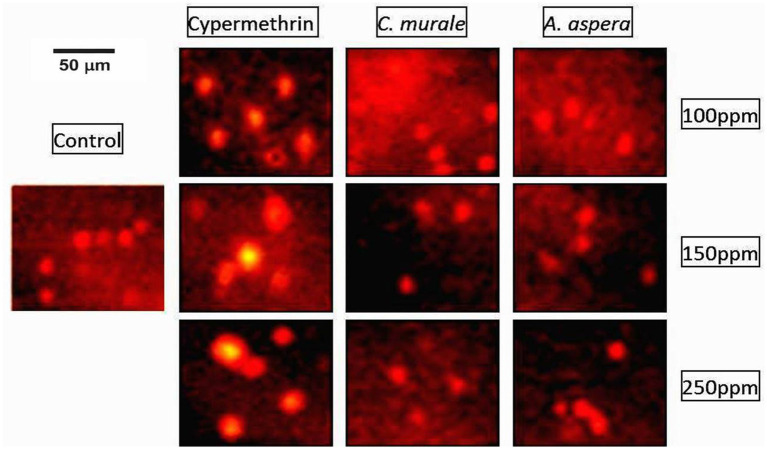
Comet assay for genotoxic effect: control, cypermethrin, and plant extracts; *C. murale* and *A. aspera* groups at different doses (28 days).

Of genotoxic effects of cypermethrin and plant extracts, *C. murale* and *A. aspera*, cypermethrin showed a significant (*p* < 0.05) difference in comet parameters by comparing with the control group. Tail length, tail DNA, and tail movement (TM) were measured in treated and control groups after 7, 14, 21, and 28 days. Tail length was significantly (*p* < 0.05) increased, i.e., 56, 60, and 66 μm at 100, 150, and 250 ppm concentrations of cypermethrin, respectively, as compared to control group after 28 days following 7, 14, and 21 days with tail length 39, 42, and 43 μm; 43, 49, and 53 μm; and 49, 54, and 58 μm, respectively, at the concentrations of cypermethrin 100, 150, and 250 ppm, respectively. The percentage of tail DNA was significantly (*p* < 0.05) higher, i.e., 90.5, 93.5, and 98.5% at 100, 150, and 250 ppm of cypermethrin, respectively, as compared to control group after 28 days following 7, 14, and 21 days with percentage of tail DNA (60.23, 63.34, and 68.87%), (68.38, 70.52, and 86.51%), and (61.68, 88.06, and 93.46%), respectively, at different concentrations of cypermethrin (100, 150, and 250 ppm), respectively. Tail movement (TM) was significantly (*p* < 0.05) increased, i.e., 15.78, 16.55, and 25.56 μm at 100, 150, and 250 ppm concentrations of cypermethrin, respectively, as compared to control group after 28 days following 7, 14, and 21 days with tail movement (7.23, 9.16, and 9.74 μm), (9.74, 12.26, and 13.74 μm), and (12.72, 14.04, and 15.43 μm), respectively, at different concentrations of cypermethrin (100, 150, and 250 ppm), respectively. All the comet parameters were significantly (*p* < 0.05) increased as compared to the control after 7, 14, 21, and 28 days of exposure to different concentrations of cypermethrin, while the treatment of plant extracts showed a non-significant (*p* > 0.05) difference in comet parameters as compared to the control group (Table 7). The highest level of DNA damage was observed at the highest dose (250 ppm) of cypermethrin at 28 days (Figure 3).

**Table 7:** Genotoxic effects due to exposure to different concentrations of cypermethrin and plant extracts at various doses and post treatment intervals at 7, 14, 21, and 28 days in albino rats.

(7th day of treatment)									
Conc. of Treat-ment	Cypermethrin			*Chenopodium murale*			*Achyranthes aspera*		
	Tail. Length (μm)	Tail DNA (%)	T. M (μm)	Tail. Length (μm)	Tail DNA (%)	T. M (μm)	Tail. length (μm)	Tail DNA (%)	T. M (μm)
*F*-value	(*F* = 27.11)	(*F* = 48.70)	(*F* = 17.00)	(*F* = 10.23)	(*F* = 10.57)	(*F* = 10.35)	(*F* = 10.27)	(*F* = 10.54)	(*F* = 10.28)
Control	14 ± 3.34a	16.18 ± 2.41a	2.26 ± 0.52a	14 ± 3.34a	16.18 ± 2.41a	2.26 ± 0.52a	14 ± 3.34a	16.18 ± 2.41a	2.26 ± 0.52a
100 ppm	39 ± 2.53b	60.23 ± 1.52b	7.23 ± 0.34b	15 ± 2.46a	16.43 ± 1.57a	2.68 ± 0.46a	14.85 ± 1.82a	16.84 ± 0.85a	2.58 ± 0.32a
150 ppm	42 ± 1.53b	63.34 ± 4.34ab	9.16 ± 0.53bc	16 ± 3.77a	18.38 ± 1.46a	2.84 ± 0.57a	16 ± 1.71a	18.43 ± 1.21a	2.46 ± 0.61a
250 ppm	43 ± 3.53b	68.87 ± 3.37bc	9.74 ± 0.34bc	16 ± 0.89a	18.82 ± 1.38a	3.05 ± 0.66a	16.63 ± 1.53a	18.69 ± 1.28a	2.74 ± 0.42a
(14th day of treatment)									
*F*-value	(*F* = 13.8)	(*F* = 15.25)	(*F* = 19.82)	(*F* = 10.31)	(*F* = 10.52)	(*F* = 10.29)	(*F* = 10.34)	(F = 10.57)	(*F* = 10.26)
Control	15 ± 2.41a	16.84 ± 1.68a	2.58 ± 0.48a	15 ± 2.41a	16.84 ± 1.68a	2.58 ± 0.48a	15 ± 2.41a	16.84 ± 1.68a	2.58 ± 0.48a
100 ppm	43 ± 4.53b	68.38 ± 3.78c	9.74 ± 1.05b	15.74 ± 1.35a	16.18 ± 1.04a	2.61 ± 0.28a	15.45 ± 1.24a	16.72 ± 1.26a	2.83 ± 0.32a
150 ppm	49 ± 3.17bc	70.52 ± 1.58c	12.26 ± 2.34bc	16.68 ± 1.58a	17.08 ± 1.21a	2.85 ± 0.79a	16.24 ± 1.34a	16.88 ± 1.63a	2.79 ± 0.29a
250 ppm	53 ± 2.45c	86.51 ± 3.06d	13.74 ± 1.56bc	16.96 ± 0.89a	17.13 ± 1.62a	3.08 ± s0.84a	17 ± 0.93a	17.62 ± 1.08a	2.91 ± 0.46a
(21st day of treatment)									
*F*-value	(*F* = 14.3)	(*F* = 15.56)	(F = 10.4)	(F = 10.28)	(*F* = 10.47)	(*F* = 10.21)	(*F* = 10.24)	(*F* = 10.43)	(*F* = 10.19)
Control	13.61 ± 2.84a	16.32 ± 1.73a	2.43 ± 0.39a	13.61 ± 2.84a	16.32 ± 1.73a	2.43 ± 0.39a	13.61 ± 2.84a	16.32 ± 1.73a	2.43 ± 0.39a
100 ppm	49 ± 2.43b	71.68 ± 1.28b	12.72 ± 1.45b	14.63 ± 1.46a	16.84 ± 0.43a	2.65 ± 0.56a	13.78 ± 1.89a	16.28 ± 0.36a	2.47 ± 0.54a
150 ppm	54 ± 1.89c	88.06 ± 1.89c	14.04 ± 1.31b	13.82 ± 1.25a	16.48 ± 0.66a	2.85 ± 0.69a	14.82 ± 1.49a	16.73 ± 0.57a	2.67 ± 0.46a
250 ppm	58 ± 1.43c	93.46 ± 2.43d	15.43 ± 1.25c	15.62 ± 1.96a	17.38 ± 1.27a	2.93 ± 0.24a	15.03 ± 1.69a	16.83 ± 1.63a	2.88 ± 0.33a
(28th day of treatment)									
*F*-value	(*F* = 14.9)	(*F* = 16.37)	(*F* = 6.23)	(*F* = 14.37)	(*F* = 16.41)	(*F* = 6.25)	(*F* = 14.40)	(*F* = 16.44)	(*F* = 6.22)
Control	14.88 ± 1.24a	17.04 ± 1.49a	2.39 ± 0.39a	14.88 ± 1.24a	17.04 ± 1.49a	2.39 ± 0.39a	14.88 ± 1.24a	17.04 ± 1.49a	2.39 ± 0.39a
100 ppm	56 ± 3.87b	90.5 ± 1.35c	15.78 ± 1.68b	15.67 ± 1.45a	17.66 ± 0.83a	2.58 ± 0.48a	15.87 ± 1.42a	18.07 ± 1.68a	2.74 ± 0.38a
150 ppm	60 ± 2.97bc	93.5 ± 3.78c	16.55 ± 1.83b	16 ± 1.69a	17.49 ± 0.38a	2.73 ± 0.53a	16.07 ± 0.52a	18.58 ± 1.72a	2.46 ± 0.44a
250 ppm	66 ± 1.78c	98.5 ± 1.27 cd	25.56 ± 3.62c	16.71 ± 2.06a	18.76 ± 1.43a	2.89 ± 0.21a	16.38 ± 1.32a	18.21 ± 1.27a	3.05 ± 0.86a

*p* < 0.05, Significant; same letters among concentrations indicate a non-significant difference.

## 4 Discussion

Plant-based extracts have been reported with a variety of properties they exhibit against various insect pests, i.e., entomocidal effect, insect repellants, insect attractants, anti-feedants, oviposition inhibition, enzyme inhibition, growth regulation, and impact on adult emergence (19, 20, 23, 28, 30, 43). Because of their complex chemical composition, it is comparatively difficult for any pest to develop resistance (43). Hence, given their eco-friendly nature and keeping in view the issues of insecticide resistance and human health concerns, it is evident that plant-derived biopesticides have a promising future (43, 44).

Indigenous weed plants, *A. aspera* and *C. murale,* have previously been reported to be very effective against many insect pests by our laboratory (19, 20, 23, 28, 30). So, to further evaluate whether these plants have some adverse effects on mammalian models, the present study was performed. Regarding cypermethrin, it has been reported by Pakistan Crop Protection Chemicals Market that it leads at the top among synthetic insecticides across Pakistan. It is estimated that it would grow from 251.3 million USD in 2025 to ~300 million USD by 2030 (31). Therefore, cypermethrin was used as a reference treatment. Different parameters, including enzyme activity, level of hepatic enzymes in blood serum, damage to liver tissues (histopathology), and genotoxic impact (comet parameters), were studied. Cholinesterases are reported with special concern in the toxicology of all pesticides affecting the nervous system; hence, modulation of AChE is useful as a marker for hepatotoxicity (36). Thus, AChE and AlkP enzymes, which are modulated in insects, were also focused on in the present study (19, 20, 28, 30, 33, 36). AChE activity was found to be significantly decreased with cypermethrin in the current study, which is in agreement with the previous findings of Kašuba et al. (36) in liver and kidney tissues for 28 days. Furthermore, consistent with Hadi and Yassin (38) and Veerappan et al. (39), AlkP activity was significantly increased with cypermethrin. However, the treatment with plant extracts showed a non-significant difference in the activity of AChE and AlkP. The significant change in the activity of enzymes against cypermethrin showed hepatotoxicity, while no change in liver tissue was found with both plant extracts. It is worth mentioning that these plants have already been reported to be toxic to targeted insects (19, 20, 23, 28, 30). Similarly, liver enzymes in serum, i.e., S. bilirubin, SGOT, SGPT, and A. phosphatase were found significantly higher with cypermethrin, and both *C. murale* and *A. aspera* showed a non-significant difference. This is in contrast with some previous studies wherein plant extracts caused changes in hepatic enzymes and altered the biochemical parameters in rats (45–47). Overall, the current biochemical findings confirm liver damage in albino rats due to cypermethrin, and no effect with both *C. murale* and *A. aspera* verify their non-toxicity to albino rats. Furthermore, histological analyses of liver and kidney tissues are in line with those of Seven et al. (33) showed damage in tissues of both organs at all concentrations of cypermethrin. These results confirm that the extracts from *C. murale* and *A. aspera* are cytologically and biochemically not toxic to albino rats. In consistent with the results of (21, 22), the level of creatinine, urea, uric acid, and hematological parameters (Hb, TLC, RBCs, HCT, MCH, MCHC, Platelets, Neutrophils, Lymphocytes, Monocytes, and Eosinophils) were also altered significantly with the treatment of cypermethrin.

Resistance against synthetic pesticides has previously been reported in many studies (18, 48, 49); thus, the genotoxicity of synthetic pesticides is an indication of subsequent resistance (33, 36). DNA damage associated with low residual levels of synthetic pesticides can induce mutagenicity, genotoxicity, carcinogenicity, and subsequent genetic disorders, bone marrow disorders, birth defects, impotence, and infertility or sterility in mammals (10, 12, 14, 33, 36). The comet assay performed in the present study showed that all the comet parameters (tail length, tail DNA, and tail movement) were significantly increased, which also confirmed the genotoxic effects of cypermethrin, whereas both *C. murale* and *A. aspera* showed non-significant differences in comet parameters as compared to the control. These results are supported by previous studies wherein cypermethrin induced genotoxicity in rats while the plant extracts showed the protective effects on these genetic alterations (10, 14). In a nutshell, both plants had non-significant differences comparable to the control for all toxicological parameters, which indicates that they are not toxic to rats and thereby to the vertebrates as compared to the synthetic insecticide, the cypermethrin. These findings suggest that these weed plants have the potential to be used as biopesticides for future Integrated Pest Management (IPM) programs.

## 5 Conclusion

Being a rapid insect pest controlling agent worldwide, synthetic pesticides pose a great risk to humans and the environment, which direly underscores the necessity of an alternative measure. Hence, plant-based biopesticides provide a good opportunity to be incorporated into pest management programs. Previously, we showed that leaf extracts of *Chenopodium murale* and *Achyranthes aspera* had an insecticidal potential, and to move ahead from a vertebrate safety viewpoint, both plant extracts were employed to evaluate biochemical, histopathological, and genotoxic effects in albino rats. Cypermethrin was used as a reference treatment, which showed congestion in the central vein, hemorrhage in hepatic tissues, and necrosis of liver tissues; while in kidney tissues, hemorrhage was attenuated by degenerated inflammatory cells, edema, and shrinkage and rupturing of glomeruli, necrosis of renal tubules, fibrosis, and swelling in Bowman’s capsule were observed. With plant extracts, no physical signs of toxicity and alteration in the micro-arrangement of the kidney were observed. The enzymes, Acetylcholinesterase (AChE) and Phosphatase, showed significant results with cypermethrin, while non-significant results with plant extracts. Furthermore, genotoxicity through the comet assay revealed no changes with both plant extracts. Moreover, no significant change was observed in blood parameters with plant extracts. Overall, both studied plants showed a non-significant difference to the control for all parameters, which is an indication of their non-toxicity to rats, and, thereby, it was concluded that these weeds are not toxic to vertebrates. Thus, focus should be given to develop a plant-extract-based biopesticide from these plants and incorporate it in IPM programs to avoid environmental and human health issues in future.

## Data Availability

The original contributions presented in the study are included in the article/Supplementary material, further inquiries can be directed to the corresponding author.
