# Supplementary material for: Evaluation of biochemical, histopathological, hematological, and genotoxic effects of some indigenous weed plant extracts in albino rats toward a natural and safe alternative to synthetic insecticides
Source: Front Vet Sci. 2026 Feb 5;13:1694297. doi: 10.3389/fvets.2026.1694297 (PMC12916361; doi:10.3389/fvets.2026.1694297)
Supplement: Supplementary file 1 [file Table_1.docx]

Supplementary Table 1: Means comparison of the data pertaining to the effect of ethanolic plant extract at various doses and post treatment intervals on blood parameters of Albino rat at day7

| **Parameters** | **Reference Value** | **Control group** | **Cypermethrin** | | | ***Chenopodium murale*** | | | ***Achyranthes aspera*** | | |
| --- | --- | --- | --- | --- | --- | --- | --- | --- | --- | --- | --- |
|  |  |  | **100ppm** | **150ppm** | **250ppm** | **100ppm** | **150ppm** | **250ppm** | **100ppm** | **150ppm** | **250ppm** |
| Haemoglobi (g/dl)  T.L.C (X109/L)  R.B.C(x1012/L)  HCT (%)  MCV (fi)  MCH (pg)  MCHC (%)  Platelets(x109/L)  Neutrophils%  Lymphocytes%  Monocytes%  Eosinophils% | 14-18  4.0-11.0  4.6-6.0  40-54  80-93  26-32  32-36  150-400  35-65  23-53  2-11  1-4 | 14.0±0.05a  5.8±0.57a  7.20±0.57a  37.9±0.57a  80.1±0.05a  25±0.05a  36±0.05a  850±0.57a  38±0.57a  32±0.57a  2±0.57a  2±0.57a | 11.50±0.01b  11.50±0.5b  6.88±0.2a  71±0.5b  101±0.1b  51±0.5b  41±0.1b  501±0.5b  71±0.5b  47±0.5b  16±0.2b  11±0.2b | 12.50±0.1b  12.5±0.2b  5.30±0.5b  62±0.05b  102±0.1b  51±0.2b  41±0.3b  551±0.5b  73±0.2b  59±0.1c  26±0.3c  11±0.5b | 11.33±0.5b  16.7±0.1c  5.33±0.5  73±0.2b  102±0.1b  51±0.2b  43±0.5b  601±0.2c  72±0.1b  61±0.2d  27±0.5c  12±0.5b | 13.86±0.5a  5.60±0.5a  6.20±0.5a  36.5±0.5a  80.0±0.5a  25.0±0.5a  33.8±0.5a  871±0.5a  38.5±0.5a  41.0±0.5a  3.6±0.5a  2.7±0.5a | 15.50±0.3  a  4.63±0.5a  5.00±0.5a  37.3±0.5a  81.3±0.5a  26.8±0.5a  31.0±0.5a  881±0.5a  41±0.3a  43±0.5ab  3.9±0.5a  2.6±0.5a | 15.±0.3a  5.40±0.5a  6.70±0.5a  36.0±0.5a  81±0.5a  28±0.05a  32.9±0.5a  801±0.5a  44.0±0.5a  41±0.5ab  3.8±0.5a  3.1±0.5a | 15.6±0.5a  5.6±0.3a  4.9±0.5a  36.3±0.5a  81±0.5a  23.8±0.5a  20.8±0.5a  835±0.5a  51±0.5a  46±0.5b  5.5±0.5a  1.7±0.5a | 14.30±0.3a  5.2±0.8a  5±0.5a  36.1±0.5a  83±0.5a  26.7±0.5a  23±0.5c  755±0.5b  52±0.3a  50±0.5a  5.6±0.5a  1.8±0.5a | 14.00±0.05a  5.0±0.5a  5.1±0.5a  36.9±0.5a  86±0.5a  28.9±0.5a  23.3±0.5c  744±0.5b  55±0.5a  54±0.5a  5.9±0.5a  2.0±0.5a |

Supplementary Table 2: Means comparison of the data pertaining to the effect of ethanolic plant extract at various doses and post treatment intervals on blood parameters of Albino rat at day 21

| **Parameters** | **Reference Value** | **Control group** | **Cypermethrin** | | | ***Chenopodium murale*** | | | ***Achyranthes aspera*** | | |
| --- | --- | --- | --- | --- | --- | --- | --- | --- | --- | --- | --- |
|  |  |  | **100ppm** | **150ppm** | **250ppm** | **100ppm** | **150ppm** | **250ppm** | **100ppm** | **150ppm** | **250ppm** |
| Haemoglobin(g/dl)  T.L.C(X109/L)  R.B.C(x1012/L)  HCT(%)  MCV (fi)  MCH (pg)  MCHC (%)  Platelets(x109/L)  Neutrophils%  Lymphocytes%  Monocytes%  Eosinophils% | 14-18  4.0-11.0  4.6-6.0  40-54  80-93  26-32  32-36  150-400  35-65  23-53  2-11  1-4 | 14.0±0.5a  5.8±0.5a  7.20±0.5a  37.9±0.5a  80.1±0.5a  25±0.5a  36±0.5a  850±0.5a  38±0.5a  32±0.5a  2±0.5a  2±0.5a | 12.13±0.1ab  9.13±0.3b  5.72±0.5b  72±0.1b  95±0.1b  50±0.5b  40±0.5b  500±0.2b  70±0.02b  57±0.01b  15±0.5b  10±0.5b | 11.50±0.1b  11.50±0.5bc  5.38±0.5b  74±0.5b  100±0.1c  52±0.2b  42±0.3b  550±0.5b  72±0.03b  58±0.01b  27±0.3c  12±0.5b | 10.33±0.2b  15.70±0.1c  4.33±0.5b  78±0.2b  103±0.1c  52±0.2b  43±0.5b  600±0.2c  73±0.01b  60±0.02bc  28±0.5c  13±0.5b | 16.26±0.6a  5.50±0.5a  7.50±0.5a  39.1±0.5a  85.1±0.5a  27.01±0.5a  34.90±0.5a  730±0.5a  42.04±0.5a  39.00±0.5a  3.0±0.5a  2.2±0.5a | 16.00±0.5a  5.30±0.5a  7.20±0.5a  38.9±0.5a  85.6±0.5a  27.05±0.5a  34.10±0.5a  750±0.5a  45.09±0.5a  42.00±0.5a  4.0±0.5a  2.5±0.5a | 15.30±0.5a  5.20±0.5a  7.00±0.5a  38.1±0.5a  85.9±0.5a  27.09±0.5a  33.00±0.5a  760±0.5a  48±0.5a  42.0±0.5a  3.5±0.5a  2.9±0.5a | 12.22±0.5a  5.3±0.5a  5.2±0.5a  41.5±0.5a  82±0.5a  28.9±0.5a  25.8±0.5a  810±0.5a  57±0.5a  44±0.5a  2.1±0.5a  1.8±0.5a | 14.5±0.5a  4.0±0.5a  5.4±0.5a  41.3±0.5a  84±0.5a  30.2±0.5a  29±0.5a  750±0.5a  60±0.5a  46±0.5a  2.2±0.5a  2.5±0.5a | 13.32±0.5a  4.9±0.5a  5.9±0.05a  39.5±0.5a  86±0.5a  31.0±0.5a  30±0.5a  780±0.5a  61±0.5a  50±0.5a  2.3±0.5a  2.9±0.5a |

Supplementary Table 3: Means comparison of the data pertaining to the effect of ethanolic plant extract at various doses and post treatment intervals on blood parameters of Albino rat at day,28days.

| **Parameters** | **Reference Value** | **Control group** | **Cypermethrin** | | | ***Chenopodium murale*** | | | ***Achyranthes aspera*** | | |
| --- | --- | --- | --- | --- | --- | --- | --- | --- | --- | --- | --- |
|  |  |  | **100ppm** | **150ppm** | **250ppm** | **100ppm** | **150ppm** | **250ppm** | **100ppm** | **150ppm** | **250ppm** |
| Haemoglobin(g/dl)  T.L. C (X109/L)  R.B.C(x1012/L)  HCT (%)  MCV (fi)  MCH(pg)  MCHC (%)  Platelets(x109/L)  Neutrophils%  Lymphocytes%  Monocytes%  Eosinophils% | 14-18  4.0-11.0  4.6-6.0  40-54  80-93  26-32  32-36  150-400  35-65  23-53  2-11  1- | 14.0±0.5a  5.8±0.5a  7.20±0.5a  37.9±0.5a  80.1±0.5a  25±0.5a  36±0.5a  850±0.5a  38±0.5a  32±0.5a  2±0.5a  2±0.5a | 11.20±0.5b  10.16±0.5b  7.72±0.2ab  72±0.5b  98±0.1b  55±0.5b  43±0.1b  500±0.5b  74±0.5b  60±0.5b  19±0.2b  12±0.2b | 8.50±0.1c  13.5±0.2c  7.50±0.5b  78±0.1b  104±0.2c  58±0.3b  46±0.3b  550±0.5b  77±0.2b  62±0.1b  20±0.3b  13±0.b | 9.33±0.5c  15.7±0.1c  8.33±0.5bc  80±0.2c  105±0.1c  60±0.2c  48±0.5b  600±0.2bc  78±0.1b  64±0.2bc  28±0.5b  18±0.5 | 14.20±0.5a  6.0±0.5a  7.94±0.5a  40.80±0.5a  83.5±0.5a  24.20±0.5a  32.40±0.5a  825±0.5a  48±0.5a  45.0±0.5a  3.4±0.5a  3.3±0.5a | 14.53±0.5a  5.5±0.5a  7.45±0.1a  39.00±0.5a  86.5±0.5a  25.00±0.5a  32.50±0.5a  835±0.5a  52±0.5a  47.0±0.5a  3.5±0.5a  3.6±0.5a | 14.20±0.3a  5.3±0.5a  7.33±0.5a  38.50±0.5a  88.0±0.5a  25.90±0.5a  32.00±0.5a  850±0.5a  55±0.5a  48.0±0.5a  3.7±0.5a  3.9±0.5a | 14.70±0.5a  5.8±0.5a  7.6±0.5a  41.5±0.5a  93±0.5a  29.4±0.5a  34±0.5a  800±0.5a  63±0.5a  50±0.5a  3.4±0.5a  3.7±0.5a | 14.53±0.5a  5.5±0.5a  7.4±0.1a  40.0±0.5a  94±0.5a  29.9±0.5a  33±0.5a  790±0.5a  67±0.5a  58±0.5a  3.5±0.5a  3.9±0.5a | 14.20±0.5a  5.3±0.5a  7.3±0.5a  39.9±0.5a  95±0.5ab  31±0.5a  33.2±0.5a  750±0.5a  69±0.5ab  62±0.5b  3.7±0.5a  3.9±0.5a |
